# Association between Sarcopenia and Immediate Complications and Mortality in Patients with Oral Cavity Squamous Cell Carcinoma Undergoing Surgery

**DOI:** 10.3390/cancers14030785

**Published:** 2022-02-03

**Authors:** Chun-Hou Huang, Kun-Han Lue, Peir-Rorg Chen, Tsung-Cheng Hsieh, Yu-Fu Chou

**Affiliations:** 1 Department of Nursing, Tzu Chi University, Hualien 970374, Taiwan; hou2017@gms.tcu.edu.tw; 2 Department of Medical Imaging and Radiological Sciences, Tzu Chi University of Science and Technology, Hualien 970302, Taiwan; john.lue@protonmail.com; 3 Department of Otolaryngology, Hualien Tzu Chi Hospital, Buddhist Tzu Chi Medical Foundation, Hualien 970473, Taiwan; cprong@gms.tcu.edu.tw; 4 School of Medicine, College of Medicine, Tzu Chi University, Hualien 970374, Taiwan; 5 Institute of Medical Sciences, Tzu Chi University, Hualien 970374, Taiwan; tchsieh@gms.tcu.edu.tw

**Keywords:** oral cavity squamous cell carcinoma, sarcopenia, biomarkers, systemic immune-inflammation index, complications, survival

## Abstract

**Simple Summary:**

Surgery remains the mainstay treatment for oral cavity squamous cell carcinoma (OSCC). Up to 40% of patients with OSCC experience postoperative complications, most within the first 30 days since surgery. The early detection of postoperative complications is challenging. Sarcopenia has been shown to be a negative predictor of the surgical and oncological outcomes of patients with OSCC. The effect of sarcopenia associated with immediate complications and impaired survival after surgery for OSCC is still unknown. This study comprehensively investigated the clinical risk factors and biomarkers associated with 30-day postoperative complications and 5- and 8-year survival of patients with OSCC. Sarcopenia was an independent risk factor associated with 30-day complications, increased reoperation rate, and reduced short- and long-term overall and disease-free survival. Sarcopenia should be assessed before surgery to identify high-risk patients who require a more intensive approach to minimize complications and may be clinically helpful in tailoring treatment strategies for patients with OSCC.

**Abstract:**

Sarcopenia negatively affects oncologic outcomes. However, studies have yet to reveal whether it is associated with postoperative complications and survival among patients with oral cavity squamous cell carcinoma (OSCC). This study retrospectively enrolled 592 patients undergoing primary OSCC surgery with available computed tomography (CT) images of their third cervical vertebrae (C3) within 30 days before surgery between January 2011 and December 2020. Preoperative sarcopenia, nutritional and frailty status, tumor characteristics, comorbidities, and inflammatory markers were assessed. The outcome variables included 30-day complications based on the Buzby and Dindo classification, reoperation, 5- and 8-year overall survival, and disease-free survival. A total of 318 (53.7%) had sarcopenia; of these patients, 217 (68.2%) presented with postoperative complications, and 48 (15.1%) underwent reoperations. Sarcopenia and higher systemic immune-inflammatory index were independently associated with local to systemic 30-day complications. Sarcopenia, advanced-stage disease, and extracapsular spread were correlated with 5- and 8-year survival. The presence of sarcopenia is associated with the 30-day complications and short- and long-term survival of patients who had OSCC and underwent surgery.

## 1. Introduction

Head and neck cancer (HNC) is the sixth most common cancer worldwide and can arise in the oral cavity, pharynx, larynx, nasal cavity, paranasal sinuses, thyroid, and salivary glands [1,2]. Oral cavity squamous cell carcinoma (OSCC) accounts for 80–90% of malignancies of the oral cavity and arises in different anatomic subsites [2]. OSCC is resistant to standard chemotherapeutics, and surgery remains the main treatment [3]. Almost 40% of patients with this disease experience 30-day postoperative complications [4]. However, the early detection of postoperative morbidity and mortality is challenging. After curative treatment, patients with OSCC remain at high risks of recurrence and second primary tumors. Up to 65% of patients with HNC present with sarcopenia during the perioperative period [5].

Sarcopenia is a progressive and generalized skeletal muscle disorder that involves the accelerated loss of muscle mass and function. It can occur in various conditions, such as chronic disease, cancer, malnutrition, aging, and inactivity [6,7]. Skeletal muscle plays a critical role in regulating metabolism, inflammation, and insulin resistance [8]. Sarcopenia is associated with dose-limiting chemotherapy toxicities [9], increased postoperative complication risk [10], early treatment termination [11,12], and high mortality rate [12] in varied treatment modalities of HNC. As such, patients who have HNC and are at a high risk of adverse treatment outcomes and premature mortality should be identified. However, interpreting the relationship between preoperative sarcopenia and surgical outcomes of patients with OSCC is challenging because of inconsistent adverse event (AE) grading and heterogeneity of patients enrolled.

The tumor-node-metastasis (TNM) staging [3], pathological feature [13,14], frailty status [15], and medical comorbidities [16,17] are important for the prognosis of OSCC. Previous studies evaluated several peripheral inflammatory/immune indicators, including neutrophil-to-lymphocyte ratio (NLR) [18], platelet-to-lymphocyte ratio (PLR) [19], and systemic immune-inflammation index (SII) [20] as significant prognostic indicators of solid tumors. SII portrays tumor-promoting activities involving angiogenesis, mutagenesis, and immunosuppression [21]. A previous study [22] reported that pretreatment peripheral blood leukocyte levels are independently associated with the prognosis of patients with OSCC. However, only a few studies have focused on the importance of systemic inflammatory markers related to postoperative complications in patients with OSCC [23].

Therefore, this study aimed to comprehensively identify the characteristics and risk factors associated with 30-day postoperative complications and 5- and 8-year survival of patients with OSCC. We hypothesized that sarcopenia would be a substantial risk factor of severe complications and mortality in patients with OSCC who underwent primary surgery.

## 2. Materials and Methods

### 2.1. Patients and Study Design

The medical records of newly diagnosed patients with OSCC and those who underwent curative surgery without neoadjuvant therapy between 1 January 2011 and 31 December 2020 were retrospectively reviewed. Only patients who had available head and neck computed tomography (CT) images of the cervical vertebrae (C3) within 30 days before surgery were included in the analysis. This retrospective study was approved by the Institutional Review Board and Ethical Committee of Hualien Tzu Chi General Hospital, Buddhist Tzu Chi Medical Foundation (IRB no.: IRB109-292-B; 4 January 2021).

The following data were collected: patient demographics, Charlson comorbidity index (CCI) [24], tumor variables, five-item modified frailty index (mFI-5) [25,26], body mass index (BMI) [27], and treatment modalities. Preoperative complete blood count and albumin level were obtained within a week before surgery. NLR and PLR were calculated as the ratio of neutrophil cell and platelet counts to lymphocyte cell count, respectively. SII was determined using the following equation: NLR × platelet count. The primary study endpoints were 30-day surgery-related complications. Wound-related local complications were graded using the Buzby [28] and Dindo [29] classification to divide local and local-to-systemic complications. Major complications were defined as ≥grade III complications. The secondary endpoints were 5- and 8-year overall survival (OS) and disease-free survival (DFS).

The skeletal muscle area (SMA) was analyzed quantitatively at the C3 level of the preoperative CT image. A single axial CT slice image of the C3 level, which showed the whole vertebral arc, was selected. The SMA was quantified at the slice by applying a threshold of −29 to 150 Hounsfield units. The C3 SMA was converted using a previously described equation [30] to estimate the SMA at the third lumbar vertebral (L3) level. This value was adjusted for the patient’s height (m^2^) to obtain the lumbar skeletal muscle index (SMI) and define sarcopenia. All images were analyzed by a single researcher using the available open-source software OsiriX (Pixmeo, Geneva, Switzerland) [31] to prevent interobserver variability. Sarcopenia was defined using the following previously determined thresholds of SMI less than 46.7 cm^2^/m^2^ for men and less than 30.3 cm^2^/m^2^ for women [32].

The cutoff values of NLR, PLR, and SII were identified through receiver operating characteristic (ROC) curve analysis on major complications based on the Buzby and Dindo classification and reoperations. For 5- and 8-year OS and DFS, the cutoff values of NLR, PLR, and SII were determined using time-dependent ROC curves [33,34]. NLR, PLR, and SII were stratified into high and low levels for all subsequent analyses.

### 2.2. Statistical Analysis

Descriptive statistics such as frequency, percentage, mean, and standard deviation (SD) were provided for the sarcopenia and nonsarcopenia groups. Independent *t*-tests were used for continuous variables. The Chi-square or Fisher’s exact test was performed for comparisons between two groups for categorical variables. Logistic regression models were used to evaluate the clinical variables and postoperative complications. ROC and time-dependent ROC curve analyses of systemic inflammation indices were performed with EZR (Saitama Medical Center, Jichi Medical University, Saitama, Japan), a graphical user interface for R (The R Foundation for Statistical Computing, Vienna, Austria). A survival curve was plotted using the Kaplan–Meier method, and differences in survival between the subgroups were estimated via a log-rank test. Univariate and multivariate analyses were conducted to explore the association between the characteristics and survival outcomes of patients. *p* value < 0.05 was considered statistically significant. Statistical analysis was performed in SPSS version 25 (IBM, New York, NY, USA).

## 3. Results

### 3.1. Baseline Characteristics of Patients

This cohort study included 592 patients who met the inclusion criteria (Table 1). Sarcopenia was identified using preoperative imaging in 318 (53.7%) and 274 (46.2%) were not. The presence of sarcopenia was associated with elderly age (*n* = 62, 19.5%), a higher CCI (*n* = 225, 70.8%), advanced-stage disease (stage III and IV, *n* = 151, 47.5%), and a lower BMI (24.8 ± 3.0 kg/m^2^). There were no significant differences in sex, the prevalence of alcohol assumption, smoking and betel nut chewing, tumor characteristics, albumin, and treatment type between the groups.

### 3.2. Thirty-Day Postoperative Complications

Thirty-day major complications were reported in 145 (24.5%) and 120 (20.3%) patients according to Buzby and Dindo classification. There were 89 (15.0%) unplanned reoperations; the most common cause of reoperations was reconstructed flap-related complications (*n* = 26, 29.2%), followed by wound infection (*n* = 25, 28.0%) and necrosis (*n* = 16, 18.0%). The patients stratified by sarcopenia were subjected to a subset analysis (Table 2 and Table 3). The cutoff values identified for the inflammatory biomarkers were 3.7 for NLR, 145 for PLR, and 459 for SII of postoperative complications.

Table 4 presents the outcomes of the logistic regression of the clinical risk factors and biomarkers of postoperative complications. In accordance with the Buzby and Dindo classification, the grade of the major complications in the sarcopenia group was higher than that in the nonsarcopenia group (odds ratio (OR) = 1.74, 95% CI = 1.07–2.83; OR = 1.57, 95%CI = 1.04–2.37, respectively). The incidences of reoperation in the sarcopenia and nonsarcopenia groups were 48 (53.9%) and 41 (46.1%), respectively (OR = 1.86, 95% CI = 1.20–2.89). In multivariate analysis, sarcopenia (OR = 1.64, 95% CI = 1.10–2.42) and high SII (OR = 2.15, 95% CI = 1.40–3.30) were associated with the severity of local wound complications. Similarly, sarcopenia (OR = 1.75, 95% CI = 1.12–2.74) and high SII (OR = 1.88, 95% CI = 1.13–3.13) were higher risk factors of reoperations. Advanced-stage disease (OR = 1.69, 95% CI = 1.04–2.48), sarcopenia (OR = 1.82, 95% CI = 1.18–2.80), and high SII (OR = 1.87, 95% CI = 1.14–3.04) were associated with major local and systemic complications.

### 3.3. Survival Analysis

The median follow-up time from the date of diagnosis was 100 months (range, 6–173 months). The 5- and 8-year OS and DFS were 69% vs. 53% and 43% vs. 28%, respectively. The cutoff values of NLR, PLR, and SII as factors of the 5- and 8- year OS and DFS were stratified into low and high levels for all subsequent analyses (Supplementary Figures S1 and S2, and Table 5).

**Figure 1:**
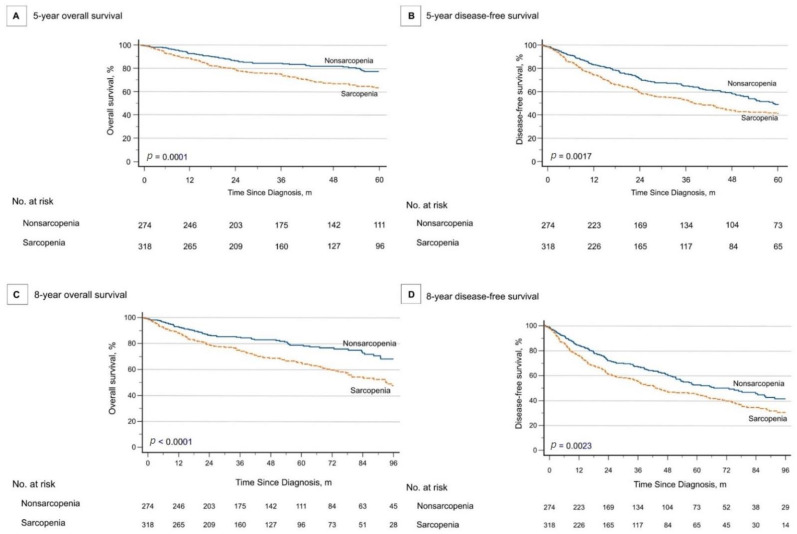
Survival of patients with and without sarcopenia: the 5-year overall survival (**A**), 5-year disease-free survival (**B**), 8-year overall survival (**C**), and 8-year disease-free survival (**D**) curves for patients according to sarcopenia status.

Univariate and multivariate Cox regression analyses predicted the 5- and 8-year OS and DFS are shown in Table 6. Multivariate analysis revealed that advanced-stage disease, perineural invasion (PNI), lymphovascular invasion (LVI), extracapsular spread (ECS), and sarcopenia were risk factors associated with 5- and 8-year OS. Higher CCI and SII were factors related to 5- and 8-year OS, respectively. Advanced-stage disease, ECS, and sarcopenia were negatively linked to 5- and 8-year DFS. The 5- and 8-year OS and DFS of patients with and without sarcopenia were subjected to Kaplan–Meier analysis (Figure 1).

## 4. Discussion

Sarcopenia [3,4,5,6,7], the frailty status [35,36], and systemic inflammatory [18,19,20,37,38] indicators are negative predictors of the surgical and oncological outcomes of patients with HNC. Based on a literature review, the present study first comprehensively identified the clinicopathological factors associated with the immediate, short-term, and long-term outcomes of patients with OSCC who underwent primary surgery. We analyzed 592 patients with OSCC. Of these patients, 318 (53.7%) had sarcopenia and underwent surgery. Within this cohort, we found that sarcopenia and high SII were significantly associated with major 30-day postoperative complications, including local-to-systemic AEs and reoperations. Sarcopenia, ECS, and advanced-stage disease were independent risk factors of 5- and 8-year OS and DFS. CCI and high SII were independent risk factors of 5- and 8-year OS, respectively.

Several studies have reported that sarcopenia is associated with surgical morbidity in patients undergoing HNC surgery. Most studies on the correlation between sarcopenia and wound complications have focused on laryngeal cancer. Furthermore, sarcopenia is an independent risk factor of the increased incidence of pharyngocutaneous fistulas and wound healing failure [10,39,40]. However, few recent reports have described the correlation between sarcopenia and postoperative AEs in patients with OSCC [41]. Consistent with previous findings [42], the results of the present study showed that the incidence rate of postoperative complications in patients with OSCC was approximately 68%. Moreover, sarcopenia is a risk factor of infection after surgery [43]. Therefore, impaired immune function is clinically correlated with sarcopenia [44]. Malnutrition is a major cause of delayed wound healing [45]. According to consensus updates about the Global Leadership Initiative on Malnutrition [46], diagnostic criteria must include an evaluation of involuntary weight loss, low BMI, and low muscle mass, in addition to etiological factors. Therefore, the early detection of OSCC with possible sarcopenia could facilitate timely education, lifestyle interventions, and more intensive monitoring.

A series of meta-analyses on different malignancies has revealed that a high pretreatment SII is strongly associated with poor progression-free survival, DFS, OS, and cancer-specific survival [20,23,47,48]. However, previous studies did not identify the role of SII in assessing postoperative complications in patients with OSCC. The results of this research showed that high SII was significantly associated with a higher risk of major postoperative complications and reoperations. Consistent with previous results [23,49,50], these results indicated that the prognosis of patients with unfavorable outcomes could be indicated more effectively by SII than by other inflammatory markers, such as NLR and PLR, because patients with a higher SII present with thrombocytosis, neutrophilia, and lymphopenia. Therefore, inflammation has a nonspecific correlation with impaired adaptive immune responses [51]. Theoretically, a qualitative abnormality in platelets that circulate and migrate to a wound microenvironment, which causes systemic abnormalities in platelet aggregation, adhesion, recruitment, and release, may directly impair anastomosis healing, release inflammatory substances, and prevent tissue repair [52,53]. Neutrophils promote inflammatory responses and secrete inflammatory mediators. By contrast, lymphocytes facilitate the regulatory reaction of the immune system that may reduce lymphocyte-mediated antibacterial cellular immune responses and participate in bacterial invasion and growth [54]. In HNC, unplanned reoperations for anastomotic failure may be attributed to thrombosis [55,56]. SII is independently associated with poor outcomes in patients with ischemic and hemorrhagic complications [57,58,59]. Thus, preoperative SII is an informative biomarker for patients with OSCC. Further prospective studies should be performed to validate the role of high SII in predicting postoperative complications for such patients, even in the absence of evident signs of infection.

Studies have been widely performed on the association of frailty, complications, and survival in HNC [35,36,60,61,62]. However, no significant differences were observed in the complications and survival of patients with and without frailty in the current study dataset. If the collection of fewer factors is likely more accessible and resource efficient, mFI-5 may be a more practical choice for predicting postoperative outcomes in clinical settings. Nevertheless, the results of the mFI-5 findings might be influenced by demographic characteristics related to comorbidities because the combined risk of major comorbidity was not assessed in the mFI-5 in our population. Moreover, comorbidity was still considered an essential feature of patients with OSCC. Patients with sarcopenia tended to be older at the time of diagnosis in this study. Sarcopenia is a common and highly prevalent clinical problem in older patients [63], and assessing elderly patients with cancer and sarcopenia could provide more personalized oncologic treatment that could ultimately improve outcomes [7,64]. By far the most important prognostic factors of OSCC include the TNM system and histopathologic features such as the PNI, LVI, and ECS of lymph nodes [13]. The presence of ECS in patients with OSCC indicates a worse prognosis, and it is associated with a higher incidence of recurrence and distant metastasis [1]. In the current study, advanced-stage disease and ECS were associated with poor short- and long-term OS and DFS. Flörke et al. analyzed 331 patients and reported advanced-stage disease resulted in remarkably higher rates of contralateral metastases [65]. However, their results of 5-year survival among patients was higher than the results of the present study. Different cultures and dietary habits could account for the difference in the survival rate between Asian and other races [66,67].

Systematic reviews [68,69] have shown that sarcopenia is associated with the poor survival outcomes of patients who have HNC and receive curative-intent treatment. However, the authors suggested that the included studies lack homogeneous tumor sites, treatment modality, outcome parameters, patient characteristics, and a large sample size, which may affect the generation of reasonable conclusions. In the present large population study, sarcopenia was independently associated with the reduced 5- and 8-year OS and DFS of patients with OSCC who underwent primary surgery. This study hypothesized that sarcopenia in patients with OSCC was amplified during the acute catabolism stage after tumor resection. Furthermore, immune senescence is correlated with changes in muscle mass-produced myokines, such as interleukin (IL)-6, IL-7, and IL-15. It also promotes natural killer (NK) cell activity and survival [44,70]. By contrast, the inflammatory cytokines tumor necrosis factor-alpha and IL-6 produced by adipocytes inhibit NK cell reactions, thereby impairing anticancer activities [70]. Notably, cachexia and sarcopenia play a role in altered protein synthesis and degradation, oxidative pathway development through muscle depletion, and systemic inflammation [71]. Therefore, sarcopenia is a negative factor of the survival outcome of patients with OSCC because of many aspects. Previously systematic reviews found that SMI at the L3 level is the most relevant indicator for detecting sarcopenia in HNC and is related to unfavorable clinical outcomes [68,69]. However, abdominal imaging is not as available in the OSCC population as head and neck imaging because positron emission tomography or abdominal CT imaging is performed in patients with a high risk of distant metastases or with advanced-stage disease. Thus, Swartz et al. [30] suggested that head and neck images can be used to measure SMI at the C3 level and indirectly calculate the L3 SMI. Additionally, many recent studies converted C3 SMI to L3 SMI and found that converted L3 measurement might still serve as a prognosticator for survival outcomes in patients with OSCC who are undergoing curative surgery [39,72,73]. However, L3 SMI estimations based on C3 SMA measurements may have calculation bias and deviate from actual measurements. As such, future prospective multicenter studies should be conducted to validate the current study findings. Although early detection of sarcopenia and systemic inflammation through routine preoperative images and peripheral blood leukocytes may allow early therapeutic intervention and develop appropriate treatment plans, the treatment of patients with sarcopenia and inflammation remains challenging [74,75].

The current study has multiple strengths. First, the study included a relatively larger CT cross-sectional area at the C3 level than previous studies [11,39,72,73] and suggested that preoperative sarcopenia defined by routinely available head and neck CT images should be considered a feasible, cost-effective, and powerful prognosticator of the incidence of immediate postoperative adverse effects (AEs) and shorter survival outcomes in patients with OSCC. Second, the assessment of postoperative AEs in patients with OSCC has not yet been thoroughly examined. The present study used the validated and standardized Buzby and Dindo classification system to record local and local-to-systemic complications, which made the study results convenient, quantifiable, and highly applicable in daily clinical practice. Third, the present study analyzed the various baseline features of radiomic, clinicopathological, and hematological parameters and prognostic biomarkers associated with clinical outcomes, which have potential individualized utility in clinical practice for patients with OSCC. Despite the inclusion of a large sample size, this study had certain limitations. First, several patients were excluded because of insufficient images used in SMI analysis, possibly introducing selection bias. Second, the study was retrospective in nature and conducted in a single Asian academic medical center. The calculated cutoff values could not be applied to a prospective study. In addition, the assessment of SII and skeletal muscle mass required prospective longitudinal studies that evaluated the dynamic changes in SII and skeletal muscle mass under a perioperative course. A risk stratification model should be established to provide a more comprehensive understanding of the influence of sarcopenia and clinicopathologic features on the complications and long-term outcomes of OSCC. Third, this study did not assess nutritional intervention effects and physical activity. Measuring muscle mass alone does not account for the loss of muscle function that occurs with sarcopenia. Functional measures of muscle strength and/or physical performance, such as walking speed test, 6 min walk test, and grip strength, should ideally be conducted for patients with sarcopenia [76]. Furthermore, patient characteristics were not subjected to matched analysis between the sarcopenia and nonsarcopenia groups because only available information was used. Although adjustments were made for several potential risk factors, our study might still have presented with residual confounding lifestyle factors (including alcohol use, betel nut consumption, and cigarette smoking).

## 5. Conclusions

Sarcopenia and SII were independently associated with the development of immediate local-to-systemic complications. Sarcopenia decreased the 5- and 8-year survival outcomes of patients with OSCC patients who underwent surgery. However, future prospective multi-institutional studies should be performed to confirm our findings. The routine assessment of sarcopenia may help clinicians develop optimal treatment strategies and more intensive approaches for this population.

## Figures and Tables

**Table 1:** Patient characteristics classified by sarcopenia before surgery.

Characteristic	Total (*n* = 592)	Sarcopenia (*n* = 318)	Nonsarcopenia (*n* = 274)	*p*
Age, years, *n* (%)				
Mean (SD)	54.2 (11)	56.8 (12)	51.3 (8)	<0.001
<65	522 (88.2)	256 (80.5)	266 (97)	<0.001
≥65	70 (11.8)	62 (19.5)	8 (3)	
Sex, *n* (%)				
Female	74 (12.5)	46 (14.5)	28 (10.2)	0.119
Male	518 (87.5)	272 (85.5)	246 (89.8)	
Alcohol, *n* (%)				
Never	152 (25.7)	74 (23.3)	78 (28.5)	0.120
Former	349 (59)	197 (61.9)	152 (55.4)	
Current	91 (15.3)	47 (14.8)	44 (16.1)	
Smoking, *n* (%)				
Never	125 (21.1)	68 (21.4)	57 (20.8)	0.879
Former	396 (66.9)	216 (67.9)	180 (65.7)	
Current	71 (12)	34 (10.7)	37 (13.5)	
Betel nut, *n* (%)				
Never	120 (20.3)	64 (20.1)	56 (20.4)	0.863
Former	319 (53.9)	175 (55)	144 (52.6)	
Current	153 (25.8)	79 (24.9)	74 (27)	
CCI, *n* (%)				
<5	211 (35.6)	93 (29.2)	118 (43.1)	<0.001
≥5	381 (64.4)	225 (70.8)	156 (56.9)	
mFI-5				
0	310 (52.4)	177 (55.7)	133 (48.5)	0.171
1	172 (29.0)	89 (28.0)	83 (30.3)	
≥2	110 (18.6)	52 (16.3)	58 (21.2)	
Cancer site, *n* (%)				
Buccal mucosa	227 (38.4)	126 (39.6)	101 (36.9)	0.722
Lower gum	158 (26.7)	81 (25.5)	77 (28.1)	
Tongue	64 (10.8)	37 (11.6)	27 (9.9)	
Lower lip	44 (7.4)	19 (6.0)	25 (9.1)	
Other sites	99 (16.7)	55 (17.3)	44 (16.0)	
Pathologic stage, *n* (%)				
1	196 (33.1)	91 (28.6)	105 (38.3)	0.033
2	138 (23.3)	76 (23.9)	62 (22.6)	
3	39 (6.6)	21 (6.6)	18 (6.6)	
4	219 (37.0)	130 (40.9)	89 (32.5)	
PNI, *n* (%)				
Negative	392 (66.2)	207 (65.1)	185 (67.5)	0.451
Positive	200 (33.8)	111 (34.9)	89 (32.5)	
LVI, *n* (%)				
Negative	379 (64)	198 (62.3)	181 (66.1)	0.338
Positive	213 (36)	120 (37.7)	93 (33.9)	
ECS, *n* (%)				
Negative	528 (89.2)	279 (87.7)	249 (90.9)	0.206
Positive	64 (10.8)	39 (12.3)	25 (9.1)	
BMI, kg/m^2^, *n* (%)				
Mean (SD)	25.3 (4.3)	24.8 (3.0)	26.9 (4.2)	<0.001
<18.5	32 (5.4)	6 (1.9)	26 (9.5)	<0.001
18.5–22.9	152 (25.6)	141 (44.3)	11 (4)	
23–24.9	130 (22)	94 (29.6)	36 (13.1)	
≥25	278 (47)	77 (24.2)	201 (73.4)	
Albumin, g/dL, *n* (%)				
Mean (SD)	3.5 (0.6)	3.4 (0.6)	3.5(0.6)	0.052
≥3.5	232 (77.3)	114 (70.8)	118 (84.8)	0.613
<3.4	68 (22.7)	47 (29.2)	21 (15.2)	
Neck dissection, *n* (%)				
No	291 (49.2)	151 (47.5)	140 (51.1)	0.381
Yes	301 (50.8)	167 (52.5)	134 (48.9)	
Free-tissue transfer, *n* (%)				
No	295 (49.8)	157 (49.4)	138 (50.4)	0.809
Yes	297 (50.2)	161 (50.6)	136 (49.6)	
Treatment type, *n* (%)				
Surgery only	282 (47.6)	155 (48.7)	127 (46.4)	0.623
Adjuvant chemo/RT	310 (52.4)	163 (51.3)	147 (53.6)	

BMI, body mass index; CCI, Charlson comorbidity index; chemo/RT, chemotherapy/radiotherapy (with or without); ECS, extracapsular spread; LVI, lymphvascular invasion; mFI-5, five-item modified frailty index; PNI, perineural invasion; SD, standard deviation.

**Table 2:** Number and grading of complications according to the Buzby and Dindo classification.

Grade	Definition	Total (*n* = 592)	Sarcopenia (*n* = 318)	Nonsarcopenia (*n* = 274)
Buzby classification (local complications)				
I	Redness, swelling, wound not opened	118 (19.9)	54 (16.9)	64 (23.4)
II	As grade I, but wound opened, dehiscence	129 (21.8)	71 (22.3)	58 (21.2)
III	Pus visible in wound	129 (21.8)	82 (25.8)	47 (17.2)
IV	Fasciitis with surgical debridement	16 (2.7)	10 (3.1)	6 (2.2)
Dindo classification (local and systemic complications)				
I	Any deviation from the normal postoperative course without the need	116 (19.6)	57 (17.9)	59 (21.5)
II	Requiring pharmacological treatment	156 (26.4)	81 (25.5)	75 (27.4)
III	Requiring surgical, endoscopic, or radiological intervention	86 (14.5)	61 (19.1)	25 (9.1)
IV	Life-threatening complication requiring intensive care management	34 (5.7)	18 (5.6)	16 (5.8)
V	Death of patient	0 (0)	0 (0)	0 (0)

**Table 3:** Causes of reoperation within 30-day in oral cavity squamous cell carcinoma undergoing surgery.

Causes	Total (*n* = 89)	Sarcopenia (*n* = 48)	Nonsarcopenia (*n* = 41)
Reconstructed flap complications	26 (29.2)	12 (25.0)	14 (34.1)
Wound infection	25 (28.0)	16 (33.4)	9 (22.0)
Necrosis	16 (18.0)	7 (14.6)	9 (22.0)
Bleeding	7 (7.9)	4 (8.3)	3 (7.3)
Flap donor site complications	8 (9.0)	5 (10.4)	3 (7.3)
Wound adhesion	7 (7.9)	4 (8.3)	3 (7.3)

**Table 4:** Logistic regression analysis between clinical risk factors and surgery-related immediate complications.

Characteristic	Buzby Classification						Dindo Classification						Reoperation					
	Univariate			Multivariate			Univariate			Multivariate			Univariate			Multivariate		
	HR	95% CI	*p*	HR	95% CI	*p*	HR	95% CI	*p*	HR	95% CI	*p*	HR	95% CI	*p*	HR	95% CI	*p*
Age (<65 vs. ≥65)	1.00	0.56–1.80	0.338				1.10	0.59–2.03	0.543				0.82	0.39–1.73	0.835			
Pathologic stage (1–2 vs. 3–4)	1.79	1.10–2.90	0.048	1.40	0.93–2.10	0.599	2.10	1.39–3.15	<0.001	1.69	1.04–2.48	0.009	2.12	1.34–3.36	0.002	1.58	1.00–2.47	0.009
PNI (negative vs. positive)	1.15	1.03–2.23	0.032	1.11	0.72–1.73	0.788	1.60	1.06–2.42	0.012	1.04	0.64–1.68	0.641	1.56	0.99–2.48	0.051			
LVI (negative vs. positive)	1.35	0.92–1.99	0.114				1.47	0.98–2.42	0.052				1.31	0.83–2.08	0.059			
ECS (negative vs. positive)	1.31	0.73–2.36	0.092				1.89	1.05–2.22	0.001	1.33	0.71–2.48	0.089	1.42	0.72–2.79	0.089			
CCI (<5 vs. ≥5)	0.62	0.38–1.01	0.051				0.94	0.62–1.43	0.262				0.98	0.61–1.57	0.619			
mFI-5 (0 vs. 1 vs. ≥2)	–	–	0.113				–	–	0.923				–	–	0.494			
Albumin (≥3.5 vs. <3..4)	0.83	0.48–1.45	0.637				0.77	0.43–1.35	0.155				0.40	0.16–1.00	0.184			
BMI (18.5–22.9 vs. <18.5 vs. 23–24.9 vs. ≥25)	–	–	0.730				–	–	0.214				–	–	0.186			
Sarcopenia (no vs. yes)	1.74	1.07–2.83	0.049	1.64	1.10–2.42	0.041	1.57	1.04–2.37	<0.001	1.82	1.18–2.80	0.001	1.86	1.20–2.89	0.041	1.75	1.12–2.74	0.044
NLR (≤3.7 vs. >3.7)	1.81	1.22–2.69	0.008	1.25	0.80–1.97	0.434	1.78	1.71–2.71	0.041	1.07	0.65–1.76	0.449	1.04	0.68–1.59	0.835			
PLR (≤145 vs. >145)	2.16	1.43–3.26	0.003	1.36	0.75–2.45	0.742	2.22	1.42–3.47	0.029	1.25	0.66–2.40	0.663	1.54	1.00–2.36	0.050			
SII (≤459 vs. >459)	2.23	1.47–3.41	<0.001	2.15	1.40–3.30	<0.001	2.37	1.49–3.76	0.005	1.87	1.14–3.04	0.042	2.38	1.40–4.04	0.004	1.88	1.13–3.13	0.012

BMI, body mass index; CCI, Charlson comorbidity index score; chemo/RT, chemotherapy/radiotherapy (with or without); Cl, confidence interval; LVI, lymphvascular invasion; mFI-5, five-item modified frailty index; NLR, neutrophil-to-lymphocyte ratio; HR, hazard ratio; PLR, platelet-to-lymphocyte ratio; PNI, perineural invasion; SII, systemic immune-inflammation index.

**Table 5:** Time-dependent receiver operating characteristic curve analysis for 5- and 8-year overall and disease-free survival.

Parameter	5-Year						8-Year					
	Overall Survival			Disease Free Survival			Overall Survival			Disease Free Survival		
	NLR	PLR	SII	NLR	PLR	SII	NLR	PLR	SII	NLR	PLR	SII
AUC	0.630	0.636	0.617	0.576	0.571	0.555	0.675	0.666	0.772	0.623	0.604	0.589
Optimal sensitivity (%)	55.1	59.8	46.2	66.0	39.8	79.0	60.0	77.6	66.8	67.0	63.0	74.0
Optimal specificity (%)	63.5	64.4	75.1	47.0	73.5	31.0	69.0	48.0	50.0	52.0	53.0	39.0
Cutoff value	3.26	148.90	337.10	4.06	171.50	348.33	3.28	150.80	337.10	4.74	171.50	360.29

AUC, area under curve; NLR, neutrophil-to-lymphocyte ratio; PLR, platelet-to-lymphocyte ratio; SII, systemic immune-inflammation index.

**Table 6:** Cox proportional hazards regression of clinical risk factors associated with survival.

Characteristic	5-Year OS			5-Year DFS			8-Year OS			8-Year DFS		
	Univariate	Multivariate		Univariate	Multivariate		Univariate	Multivariate		Univariate	Multivariate	
	*p*	HR (95% CI)	*p*	*p*	HR (95% CI)	*p*	*p*	HR (95% CI)	*p*	*p*	HR (95% CI)	*p*
Age (<65 vs. ≥65)	0.207			0.202			0.129			0.109		
Pathologic stage (1–2 vs. 3–4)	<0.001	1.88 (1.27–2.79)	0.004	<0.001	1.42 (1.10–1.83)	0.032	<0.001	1.93 (1.37–2.71)	0.001	<0.001	1.44 (1.15–1.89)	0.002
PNI (negative vs. positive)	<0.001	1.67 (1.14–2.43)	0.004	0.005	1.21 (0.91–1.61)	0.266	0.001	1.45 (1.03–2.04)	0.030	0.002	1.24 (0.92–1.74)	0.161
LVI (negative vs. positive)	<0.001	1.67 (1.12–2.47)	0.010	<0.001	1.14 (0.99–2.01)	0.052	<0.001	1.54 (1.09–2.19)	0.002	<0.001	1.29 (0.97–1.03)	0.075
ECS (negative vs. positive)	<0.001	1.80 (1.18–2.74)	0.008	<0.001	1.45 (1.04–1.75)	0.048	<0.001	1.67 (1.12–2.48)	0.018	<0.001	1.48 (1.16–2.07)	0.033
CCI (<5 vs. ≥5)	0.022	1.60 (1.12–2.28)	0.008	0.052			0.064			0.068		
mFI-5 (0 vs. 1 vs. ≥2)	0.839			0.877			0.926			0.852		
Albumin (≥3.5 vs. <3.4)	0.747			0.934			0.590			0.634		
BMI (18.5–22.9 vs. <18.5 vs. 23–24.9 vs. ≥25)	0.025	–	0.560	0.009	–	0.173	0.047	–	0.561	0.046	–	0.473
Sarcopenia (no vs. yes)	0.001	1.52 (1.08–2.13)	0.012	0.004	1.31 (1.32–1.60)	0.020	<0.001	1.76 (1.30–2.39)	0.001	0.001	1.42 (1.14–1.78)	0.003
NLR (low vs. high)	0.001	1.06 (0.69–1.63)	0.054	0.005	1.23 (0.96–1.58)	0.054	<0.001	1.10 (0.72–1.68)	0.079	0.174		
PLR (low vs. high)	<0.001	1.39 (0.98–1.96)	0.562	0.057			0.002	0.99 (0.63–1.55)	0.310	0.043	1.01 (0.80–1.29)	0.913
SII (low vs. high)	<0.001	1.20 (0.72–2.00)	0.896	0.005	0.94 (0.66–1.33)	0.912	<0.001	1.39 (1.02–1.91)	0.013	0.148		

BMI, body mass index; CCI, Charlson comorbidity index; Cl, confidence interval; DFS, disease free survival; ECS, extracapsular spread; HR, hazard ratio; LVI, lymph-vascular invasion; mFI-5, five-item modified frailty index; NLR, neutrophil-to-lymphocyte ratio; OS, overall survival; PLR, platelet-to-lymphocyte ratio; PNI, perineural invasion; SII, systemic immune-inflammation index.

## Data Availability

The data presented in this study are available on request from the corresponding author. The data are not publicly available due to the privacy and ethical restrictions.

## Supplementary Materials

The following are available online at https://www.mdpi.com/article/10.3390/cancers14030785/s1, Figure S1: Time-dependent receiver operating characteristic curves for determining optimal cutoff value of the preoperative systemic inflammatory markers for 5-year overall and disease-free survival, Figure S2: Time-dependent receiver operating characteristic curves for determining optimal cutoff value of the preoperative systemic inflammatory markers for 8-year overall and disease-free survival.
