# Supplementary material for: Association between Sarcopenia and Immediate Complications and Mortality in Patients with Oral Cavity Squamous Cell Carcinoma Undergoing Surgery
Source: Cancers (Basel). 2022 Feb 3;14(3):785. doi: 10.3390/cancers14030785 (PMC8833832; doi:10.3390/cancers14030785)
Supplement: Supplementary file 1 [file cancers-14-00785-s001.zip › cancers-1569470-supplementary.pdf]

Supplementary Online Content

Huang C-H, Lue K-H, Chen P-R, Hsieh Y-C and Chou Y-F. Association Between Sarcopenia and Immediate Complications and Mortality in Patients with Oral Cavity Squamous Cell Carcinoma Undergoing Surgery. *Cancers.*

Figure S1: Time-dependent receiver operating characteristic curves for determining optimal cutoff value of the preoperative systemic inflammatory markers for 5-year overall and disease-free survival.
Figure S2: Time-dependent receiver operating characteristic curves for determining optimal cutoff value of the preoperative systemic inflammatory markers for 8-year overall and disease-free survival.

This supplementary material has been provided by the authors to give readers additional information about their work.

Figure S1: Time-dependent receiver operating characteristic curves for determining optimal cutoff value of the preoperative systemic inflammatory markers for 5-year overall and disease-free survival.

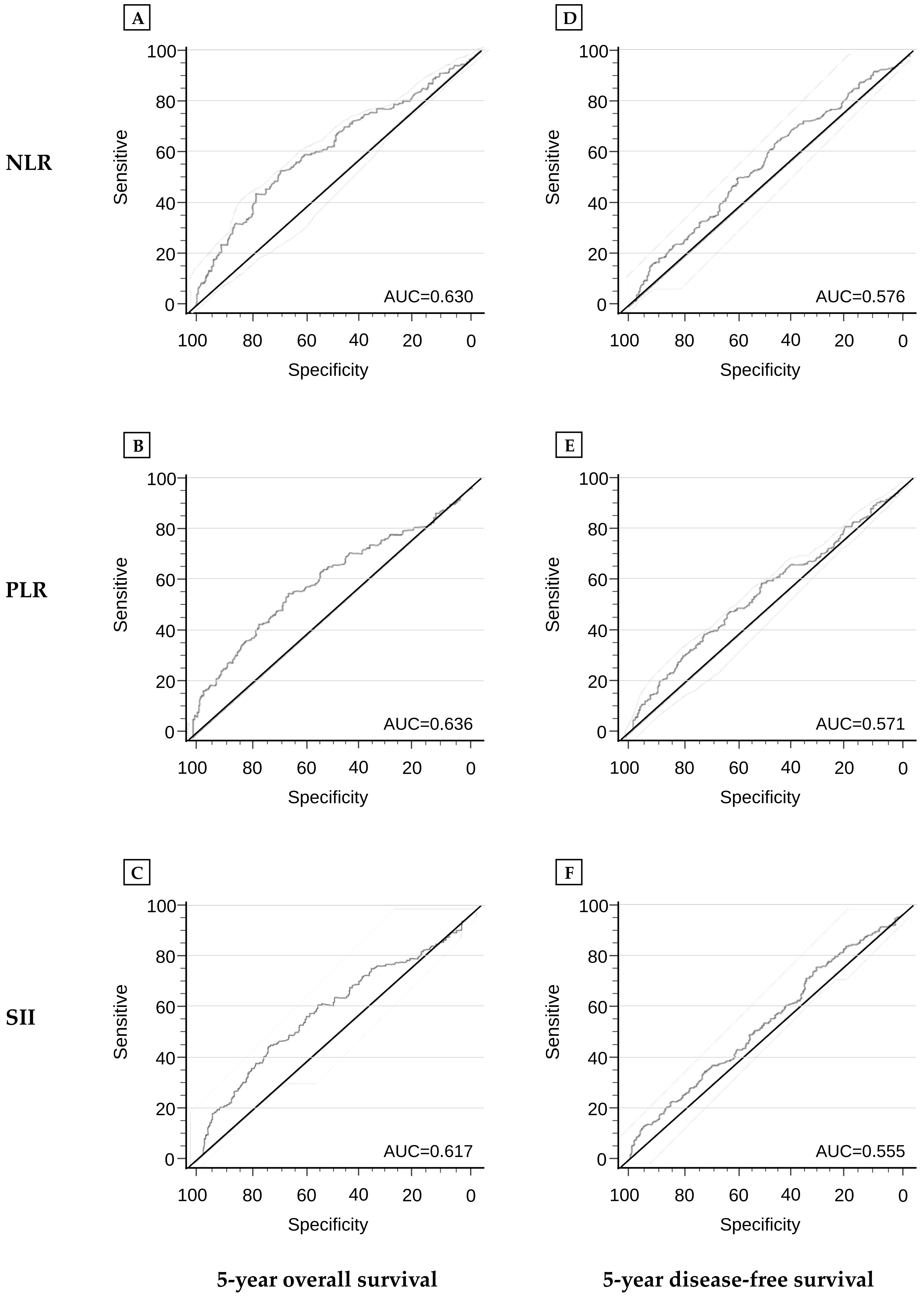

AUC indicates area under curve; NLR, neutrophil-lymphocyte ratio; PLR, platelet-lymphocyte ratio; SII, systemic immune-inflammation index.

Figure S2: Time-dependent receiver operating characteristic curves for determining optimal cutoff value of the preoperative systemic inflammatory markers for 8-year overall and disease-free survival.

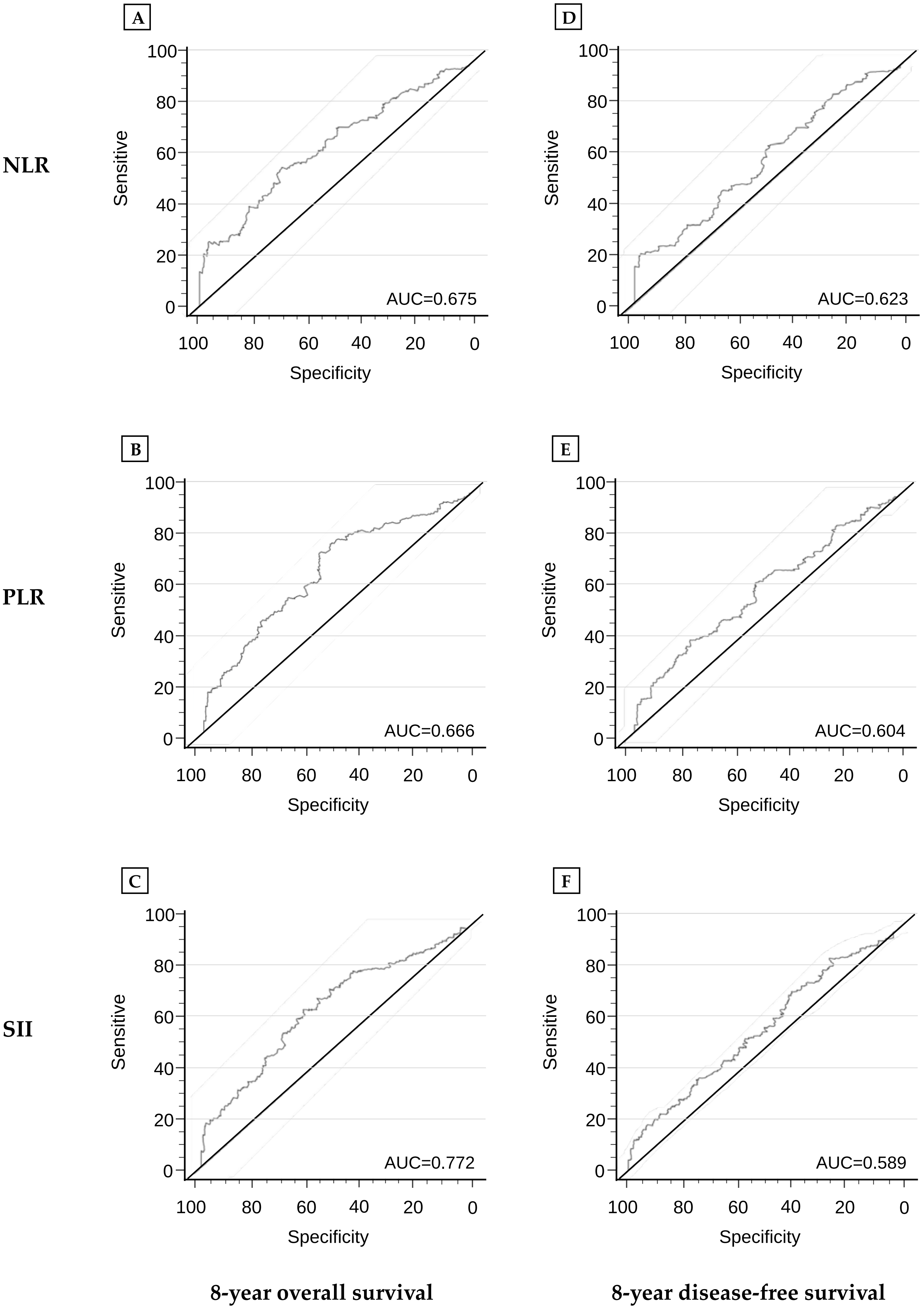

AUC indicates area under curve; NLR, neutrophil-lymphocyte ratio; PLR, platelet-lymphocyte ratio; SII, systemic immune-inflammation index.
